# An Experimental Investigation into Mechanical and Thermal Properties of Hybrid Woven Rattan/Glass-Fiber-Reinforced Epoxy Composites

**DOI:** 10.3390/polym14245562

**Published:** 2022-12-19

**Authors:** Agustinus Purna Irawan, Paula Tjatoerwidya Anggarina, Didi Widya Utama, Najid Najid, Mohd Zulkfly Abdullah, Januar Parlaungan Siregar, Tezara Cionita, Deni Fajar Fitriyana, Jamiluddin Jaafar, Agung Efriyo Hadi, Teuku Rihayat

**Affiliations:** 1 Faculty of Engineering, Universitas Tarumanagara, Jakarta 11480, Indonesia; 2 Faculty of Economics and Business, Universitas Tarumanagara, Jakarta 11480, Indonesia; 3 School of Mechanical Engineering, Engineering Campus, Universiti Sains Malaysia, Nibong Tebal 14300, Malaysia; 4 Faculty of Mechanical & Automotive Engineering Technology, Universiti Malaysia Pahang, Pekan 26600, Malaysia; 5 Faculty of Engineering and Quantity Surveying, INTI International University, Nilai 71800, Malaysia; 6 Department of Mechanical Engineering, Universitas Negeri Semarang, Kampus Sekaran, Semarang 50229, Indonesia; 7 Faculty of Mechanical and Manufacturing Engineering, Universiti Tun Hussein Onn Malaysia, Parit Raja 86400, Malaysia; 8 Mechanical Engineering Department, Faculty of Engineering, Universitas Malahayati, Jl. Pramuka No. 27, Kemiling, Bandar Lampung 35153, Indonesia; 9 Department of Chemical Engineering, Politeknik Negeri Lhokseumawe, Lhokseumawe 24301, Indonesia

**Keywords:** rattan strips, woven, glass fiber, hybrid, composite

## Abstract

The investigation of hybrid, woven, natural fiber-reinforced polymer composites as a substitute reinforcement for fiber polymer composites has recently caught the interest of academics, industry, and researchers. Woven, natural fiber composites have been implemented in many different applications, including parts for automobiles, household items, flooring, aerospace, and ballistic materials. Therefore, this research seeks to establish the thermal and mechanical characteristics of composites made from rattan strips (RS) and glass fiber (GF)-reinforced epoxy resin (ER). Other than that, the impact of layering configurations with respect to the thermal and mechanical characteristics of the RS and GF will be determined. Hand lay-up and a hydraulic press machine produce hybrid, woven RS and GF laminates. The hybrid composite’s mechanical properties will be investigated using impact, tensile, and flexural tests. The hybrid woven of the GF/RS/RS/RS/GF composite sequence demonstrated the highest mechanical properties in comparison to other sequences. The increase from one to three layers of RS in the core layer of GF hybrid composites enhanced the flexural, impact, and tensile properties. In addition, the hybridization of rattan and GF is more thermally stable, as recorded by the high decomposition temperature. As a finding of the research, the woven RS and GF hybrid is a potential material for automotive applications such as car bumpers, for example.

## 1. Introduction

**Figure 1:**
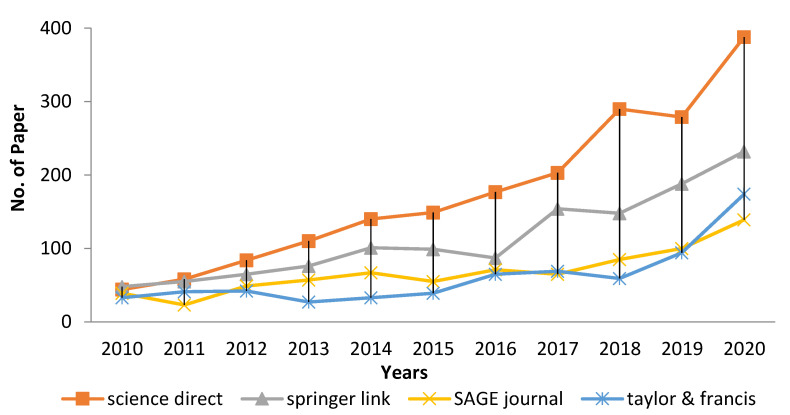
The number of research articles from 2010 to 2020 using the keyword woven, natural fiber-reinforced polymer composite (W-NFRPC).

In fiber-reinforced polymer composites, natural fibers exist in various forms, including continuous, random, and fabric [1]. According to Aisyah et al. (2021), woven composites comprise textiles with the maximum stability and flexibility [2]. In addition, woven fabrics are desirable as reinforcements due to their high level of integrity and conformability [3,4]. The purpose of woven textiles is to suit the needs of their intended receiver. During the previous 10 years, the utilization of woven, natural fibers as a substitute for synthetic materials has increased in importance, accompanied by rapid development [5,6]. The increasing number of investigations into woven, natural fiber-reinforced polymer composite (W-NFRPC) materials, as depicted in Figure 1, indicates that the appealing qualities of composite materials have captured the attention of numerous researchers, academicians, and companies around the globe.

The qualities of finished woven fiber-composite products are controlled by variables such as fiber type, fiber content, fiber layering, fiber stacking sequence, and fiber moisture content, all of which have a substantial effect on W-NFRPC processing. There are several factors that affect the mechanical and physical characteristics of woven, natural fiber composites, and multiple pieces of research have reported their outcomes [7,8]. Researchers have undertaken several initiatives to enhance the mechanical properties of W-NFRPC, for example, by the use of a hybridization method between natural woven fiber and synthetic fiber. The performance of hybrid composites can be considered as the weighted sum of the individual components, with the purpose being to find a more positive balance between the inherent benefits and drawbacks of each component. When utilizing a hybrid composite consisting of two or more distinct types of fiber, the benefits of one type of fiber may enhance what is lacking in another type of fiber. As a direct result of this, it is possible, via the utilization of an appropriate material design, to achieve a good balance between cost and performance [9,10]. When compared to the results obtained from hybridizing a natural fiber with a natural fiber in a composite, the hybridized composite of high-strength synthetic fibers, for example, carbon fiber and glass fiber (GF), with a natural fiber produced excellent mechanical properties [11].

There are a growing number of reviews and research articles investigating the effects of hybridization with respect to the performance of synthetic fiber and W-NFRPC for automotive applications, such as automobile bumper beams [12,13,14,15,16,17,18]. The primary purpose of a bumper system is to protect the vehicle’s body and passengers from collision damage. The main components of a front bumper system are the bumper beam, the absorber, and the fascia [19]. Note that most polymer composites that were created for bumper beam materials were studied for the material’s mechanical properties, which include its flexural properties (modulus and strength), tensile properties (modulus and strength), and impact characteristics [10].

Davoodi et al. (2010) developed and tested the mechanical properties of a synthetic GF and hybrid kenaf fiber-reinforced epoxy composite for passenger automobile bumper beams. Utilizing a sheet molding compound (SMC) method, hybrid materials were produced. The results of their investigation indicate that the flexural and tensile properties of hybrid kenaf/GF are greater than those of conventional bumper beam materials, such as glass mat thermoplastics (GMT). Meanwhile, the impact energy of hybrid kenaf/GF was determined to be 26 J/m, almost half that of the typical GMT [16]. Other than that, the mechanical properties of jute and GF composites for car bumper beams were evaluated by Olorunnishola et al. (2018). Hand lay-up composites were created by laying the composites on the bumper mold along with commercial-grade polypropylene spraying. The samples underwent a 24 h post cure, and the surface was finished with a grinding wheel. Subsequently, three examples were made using 40% wt GF, 40% wt jute fiber, and 10% wt. GF with 30% wt. jute fiber. Apart from that, a maleated polypropylene (MAPP) compatibilizer was used to provide bonding between the polymer and the fibers. A universal testing machine was utilized to assess hardness and impact qualities, whereas a Charpy V-notch machine was employed to identify impact energy. Moreover, the hybrid model outperformed the commercial bumper material in terms of hardness and impact resistance [20].

Maisuriya et al. (2020) investigated the flexural and tensile properties of banana fiber/glass-reinforced polyester hybrid composites manufactured via the hand lay-up process. According to the study’s findings, adding GF to composite materials improves both the flexural and tensile properties of the materials. Per the results, the flexural modulus and strength were both raised when the percentage of GF increased from 0 to 30 wt.%, increasing the MPa from 41.1 to 168.3 and the GPa from 1.3 to 5.7, respectively. Similarly, hybrid composites consisting of 20% banana and 20% GF achieved maximum values of 152.3 MPa and 4.0 GPa for their tensile strength (TS) and modulus, respectively [21]. Santhanam et al. (2021) investigated the impact of a five-layer stacking sequence of hybrid banana and GF-reinforced epoxy composite. The TS of the epoxy reinforced with banana fiber was 37.2 MPa, but hybridization with GF enhanced the TS to 84 MPa. The TS of specimens G-B-B-B-G and B-G-B-G-B were closer to each other, as were the tensile strengths of specimens B-G-G-G-B and G-B-G-B-G, indicating that variation in the stacking sequence has little impact on the tensile strength. Furthermore, the impact test findings show that the stacking sequence has no effect. However, hybridization of the banana fiber composite yielded higher values compared to pure banana fiber composites. The pure GF-reinforced epoxy composite possesses the highest impact strength value. Hence, hybridization enhanced the value of impact strength by 54% [22].

On the other hand, Gujjala and colleagues (2014) investigated the mechanical properties of a hybrid jute/glass-reinforced epoxy composite. Various stacking sequences of woven hybrid composite laminates were arranged by utilizing the hand lay-up technique. Furthermore, the modulus and TS of glass/jute/jute/glass (GJJG)-staked hybrid jute and glass are greater than those of GJGJ, JGGJ, and pure JJJ. The incorporation of GFs substantially raises the TS of pure jute laminate by 66% (GJJG), 51% (GJGJ), and 41% (JGGJ). The GJGJ hybrid composite established the greatest flexural modulus and flexural strength [23]. Alternatively, Ramnath et al. (2013) fabricated and evaluated the mechanical properties of an abaca–jute–GF-reinforced epoxy composite. GF is laminated onto the top and bottom of the composite, improving the polish and adding strength. The tensile strengths of hybrids made of glass, banana, and jute are 85.9 MPa, 68.4 MPa, and 51.1 MPa, respectively, per Ramnath. The jute/glass hybrid composite has the lowest TS compared to the other hybrid composites. In related research, jute/GF-reinforced polyester hybrid composites exhibited the highest impact strength at 752 J/m, in comparison to jute/banana/glass hybrids’ 326 and 500 J/m values [24].

The number of layers within a woven, natural fiber laminate affects the mechanical as well as physical properties of composites made of woven, natural fiber [25]. According to Bhoopathi et al., 2014 studies, the stacking sequence affects the flexural strength result, with three layers of GF, as well as two layers of banana fiber, generating much better results than the hand lay-up of two layers of GF and three layers of banana fiber [26]. Acharya (2014) assessed the consequences of a layered stacking sequence with respect to the mechanical characteristics of treated jute and a hybrid GF-reinforced epoxy composite. A four-layered stack of glass/jute/jute/glass possessed the greatest flexural strength and TS [27]. On the other hand, Yahaya et al. (2015) examined the woven kenaf–aramid hybrid laminated composite’s mechanical properties. They reported that the four-layer hybrid composite outperformed the three-layer samples with regard to TS [28]. Similarly, Rajesh et al. (2018) found that four layers of jute basket-weaving fabric resulted in superior qualities compared to other weaves [29]. The experiment was conducted by Rajesh and Pitchaimani (2017), and Acharya (2014) utilized a four-layer stacking sequence. The results revealed that four fiber layers greatly increase the mechanical properties compared to three fiber layers [4,27].

In the aforementioned literature, the impacts of a hybrid of woven, natural fiber and synthetic fiber on the mechanical properties of an epoxy composite for bumper beam application have been reviewed. This research aims to examine the thermal and mechanical properties of hybrid, woven rattan/GF-reinforced epoxy composites. In addition, the influence of the layering number of RS and the hybrid on the tensile, impact, and flexural properties will be identified and compared. Furthermore, using thermogravimetric analysis (TGA), the excellent mechanical properties of pure and hybrid composite specimens will be observed for their thermal properties. The result of this study is expected to demonstrate the capability of fabricating competitive hybrid rattan strip with glass fiber (HRS/GF) reinforced ER composites that might be used as a replacement material for the automobile industry, particularly for bumper applications.

## 2. Materials and Methods

### 2.1. Materials

For this research, Manau rattan from Kalimantan, Indonesia, was utilized as a woven reinforcement. The outside layer (bast) of Manau rattan was cut to a diameter of 4 mm. The 30 cm × 30 cm woven rattan strips (RS) were made in the traditional way at the materials laboratory at the University of Tarumanagara in Indonesia. The bi-directional E-GF and epoxy resin (ER) for general use (Epikote 828) were bought from the IZE solution (Selangor, Malaysia). The thermal and mechanical properties of the epoxy matrix and reinforcement are presented in Table 1.

### 2.2. Fabrication of Composite Laminates

**Figure 2:**
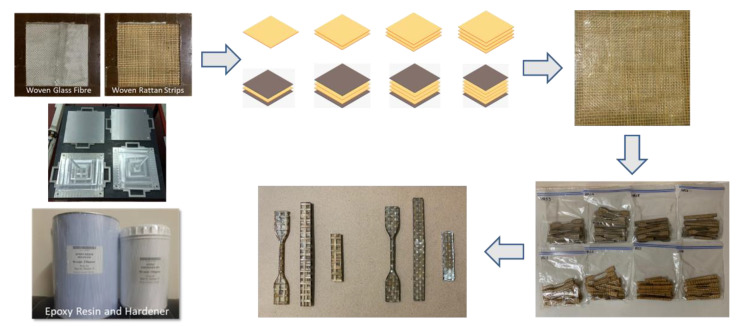
Fabrication process and specimen test for composite laminates.

The composite laminate production was accomplished by employing a hand lay-up process. In order to hinder the composite plate process from sticking to the interior surface of the mold and facilitate removal, wax or a releasing agent was sprayed on it first. After manually combining the ER and hardener in a ratio of 3:1 for 5 min, the resin was manually poured into the mold, and the RS was laid on top of the resin. This process was continued until the appropriate layer was attained, where the tolerance for fabric alignment should also be considered. For the curing procedure, the mold was laid out at room temperature for 24 h. In the desired sequence, both pure and hybrid composite laminates are listed in Table 2. The layering number of RS from 1 to 4 layers and hybrid RS with glass fiber (GF) from 3 layers to 6 layers will be examined in this study. The outer skin (top and bottom) of the woven RS was composed of 2 layers of GF. Figure 2 shows the fabrication process of composite laminates and specimen tests.

### 2.3. Tensile Test

To assess tensile properties, which include the tensile strength (TS) as well as tensile modulus (TM), specimens according to the ASTM D 638–IV standard were prepared. Using an INSTRON 3369 (INSTRON, Boston, MA, USA) universal instrumentation, a tensile test was conducted at a crosshead speed of 2 mm/min. Moreover, the TS and TM average values were determined based on seven specimens manufactured for each composite configuration type before testing.

### 2.4. Flexural Test

Utilizing the universal testing apparatus INSTRON 3369 (identical to tensile testing procedures), a three-point bending test is employed to analyze flexural strength and flexural modulus. Note that 127 × 12.7 and 3 mm were utilized to fabricate the specimen in accordance with ASTM D790-17 standard method. For each type of specimen test, seven replications were undertaken, and the average data were calculated.

### 2.5. Izod Impact Test

The Izod impact tests were conducted with Zwick Roell (Zwick Company, Ulm, Germany) and 10 J of impact energy. This test was conducted at room temperature under ASTM D256 standard method. The impact energies were determined through testing. The impact unnotched specimen dimensions were 63.5 × 12.7 × 3 mm.

### 2.6. Scanning Electron Microscopy (SEM)

In both pure and hybrid rattan strip (HRS)-reinforced epoxy composites, in order to analyze the fracture and surface morphological behavior of tensile fracture specimens, a Hitachi TM3030 Plus (Hitachi, Tokyo, Japan) scanning electron microscope was employed. Using double electrically conductive carbon adhesive tapes, the samples were first sputter-coated with a thin palladium layer to avoid surface charge before being mounted on a scanning electron microscopy (SEM) holder. The samples were then evaluated using a microscope with magnifications of 50× and 100×, having a 10 kV acceleration tension.

### 2.7. Thermogravimetric Analysis (TGA)

The thermal stability of composite filler composites with treated and untreated fillers was assessed using the Hitachi STA7200 thermogravimetric analyzer (Hitachi, Fukuoka, Japan). A 10 mg powdered sample was put in an alumina crucible before being kept in the furnace. The study was done in a controlled environment with nitrogen gas flowing at a rate of 20 mL/min. Here, 10 °C/min was the constant temperature change rate. The experiment was conducted between 30 and 700 °C.

### 2.8. Differential Scanning Calorimetry (DSC)

A differential scanning calorimetric (DSC) analysis of composites was conducted utilizing a Perkin Elmer DSC 8000 (Perkin Elmer, MA, USA). The test was conducted using 20 mL/min of nitrogen flow and a 10 °C/min heating rate up to 350 °C above room temperature.

## 3. Results and Discussion

### 3.1. Tensile Properties

**Figure 3:**
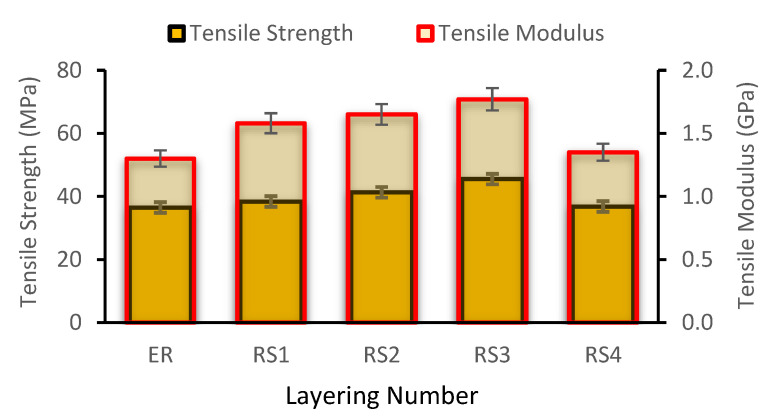
Tensile properties of epoxy composites reinforced with woven rattan strips.

Figure 3 depicts the tensile modulus (TM) as well as the tensile strength (TS) of woven rattan strips (RS) and the hybrid RS with glass fiber (GF)-reinforced epoxy resin (ER) composites. Figure 3 shows that the maximum tensile stress occurred at RS3 and gradually decreased at RS4. This is because, as the number of woven RS rises, the resin concentration decreases, leaving insufficient resin to transfer the load between fibers. In addition, the resin is insufficient to cover the fibers, yielding a drop in TS as the fiber content increases [35]. Since it is described as the slope of the stress–strain curve in the elastic deformation region, the TM of the composites, as illustrated in Figure 3, behaves similarly to the TS. When reinforced with woven RS, the elastic modulus of the pure epoxy composite rose to roughly 1.30 GPa. Similar to the tensile strength, the composite’s TM reaches its maximum value (1.77 GPa) in the RS3 specimen and decreases in the RS4 specimen. As the number of RS utilized in RS4 increased, the TM of the composite decreased. This could be attributed to the usage of rattan woven strips in RS3, which provide the strongest interfacial adhesion to the composite, resulting in an excellent load transfer [36]. However, the more rattan woven strips used in RS4 resulted in low interfacial adhesion, because the amount of ER used was insufficient to cover all areas of the woven rattan strips. Furthermore, the hemicellulose content of the composite increased as the number of woven RS used increased.

The higher the hemicellulose concentration is, the more moisture is absorbed, resulting in a significant decrement in TM as well as TS [37]. The TM illustrates a similar pattern compared to the TS discovered in research performed by Ramanaiah et al. (2013). Consequently, their results presented that the TM and TS of the composite rose as the fiber content did [38]. Per a study by Sharba et al., the TM and TS in the plain-woven, glass-reinforced unsaturated polyester (UP) hybrid composite specimens exhibited a similar pattern [39]. Agustinus and Sukania [40] found that the average TS of laminated rattan strips with ER is about 24.57 MPa. Based on their study, it can be concluded that rattan fiber is an alternative material for making car spoiler applications [40]. This study conceded that the composites’ tensile properties are determined by a variety of factors, which includes the type of reinforcement and matrix, the number of layers present, and the matrix and the fibers’ interfacial bonding [41].

**Figure 4:**
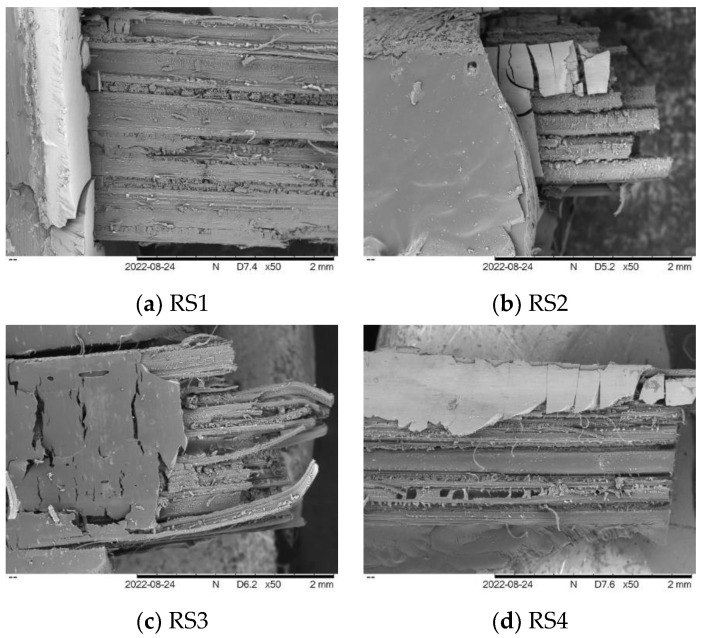
Tensile fracture specimen of epoxy composites reinforced with rattan strips.

The tensile fracture of hybrid composites was examined utilizing scanning electron microscopy (SEM). Figure 4 portrays the fractured tensile specimens of the RS composite that was examined. Note that a crucial component of composite materials is the interfacial bonding that progresses between the fiber reinforcement and the resin matrix, resulting in a higher TS of the composite. Failure mechanisms, including matrix cracking, delamination, fiber breakage, and fiber pull-out, can have an influence on the woven fiber composites’ mechanical properties [42]. Note that the micrograph of tensile fractured RS1 showed delamination on the specimen. The delamination in composites caused by inadequate interfacial bonding between the fiber and matrix resulted in a poor load transfer between the RS layers. Natural fibers are typically coated with waxy elements; this surface has low surface energy, resulting in poor adhesion to the polymer matrix [43]. Other than that, the crack-propagation mechanism is found in all the tensile fractured specimens. The fracture of the composites, which frequently comes from the creation of displacement discontinuity surfaces inside the composites, is the main cause of crack propagation. A fracture is when tension causes an object or material to split into two new pieces. Subsequently, the mechanism by which a crack spreads within composite materials by cutting through the structure’s grain grains is known as a transgranular fracture. Meanwhile, intergranular fractures develop along grain boundaries [44,45].

**Figure 5:**
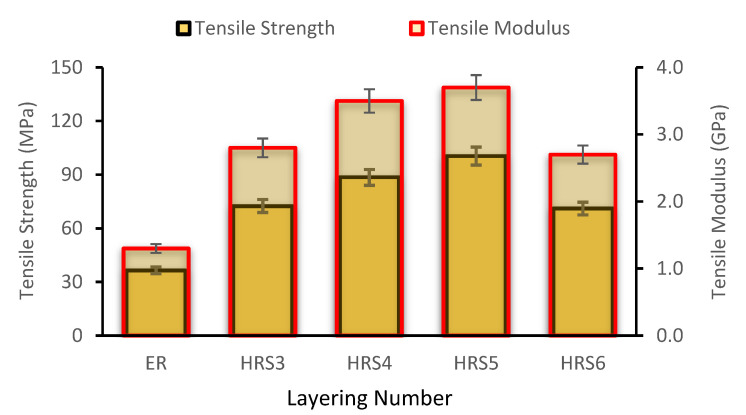
Tensile properties of hybrid, woven rattan strips with glass fiber composites.

A hybrid material combines two or more materials to produce a novel substance that possesses new characteristics and behaviors. Compared to the properties of composites made from individual fibers, the hybrid composite had superior mechanical properties [46]. Figure 5 displays the effect of one to four layers of hybrid rattan strips (HRS) mixed with two plies of GF in the outermost layers on the composite’s tensile properties, as determined by earlier research. [23,47]. Apart from that, TS and TM increased dramatically as the number of the hybrid layers was raised up to five. Here, the maximum TS and modulus of HRS5 or the combination of three layers of RS (RS3) and two plies of GF are 100.40 MPa and 3.7 GPa, respectively. The addition of two plies of GF to the outer layer of a single RS increased its TS from 36.50 MPa to 72.50 MPa. Comparing RS3 to RS1 with two layers of GF (HRS3), the hybrid composite possesses roughly 88%, which is higher in TS. In addition, the HRS3 composite’s TM is 77% more than that of three layers of RS. The addition of GF increases the strength and stiffness of hybrid composites due to their greater TS and TM, which are around 1900–2050 MPa (TS) and 72–85 GPa (TM) compared to 41.97–121.5 (TS) and 4.97 GPa (TM) for RS (see Table 1). In addition, the incorporation of GF into RS composites enhances the load-bearing ability of the hybrid composites, yielding increased strength and rigidity. This is a result of the excellent stress transfer between the RS plies and GF plies, enabling the hybrid composites to endure a higher tensile load. This pattern is consistent with the research conducted by Hariharan and Khalil [48], who worked on oil palm fiber–GF-reinforced ER. A previous study found that the TS of a single RS from four different species ranges from 464 to 603 MPa. In the meantime, the TM of a single RS is between 9.10 and 10.61 GPa [49]. Meanwhile, the tensile properties of GF are higher than those of natural fiber, as reported in previous studies [50,51]. In research comparing pure and hybrid, woven jute composites, GF had a higher specific strength than a natural fiber, and composites demonstrated more improvement when placed at the skin’s outer surface than when placed in the composites’ core [47]. Utilizing a hybrid composite made up of two or more different types of fibers might allow the advantages of one type of fiber to outweigh those of another [52].

**Figure 6:**
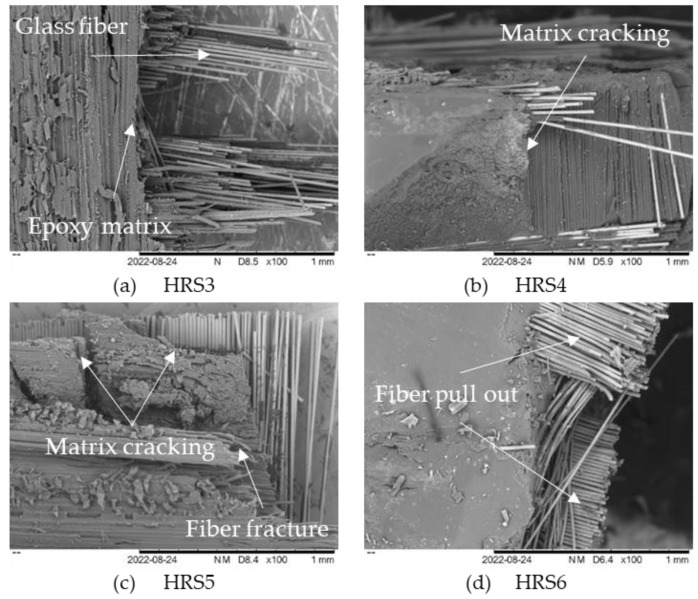
Tensile fracture specimen of hybrid rattan strips with glass fiber composites.

The HRS composite samples with various layer configurations experienced a tensile fracture, as seen by the micrograph of the fractured tensile specimen in Figure 6a–d. Observations of fractured specimens revealed that the failure of a RS composite occurs promptly with little fiber pull-out (Figure 4). Still, failure in HRS is regulated by a substantial fiber pull-out and cracking matrix, as shown in Figure 6a–d. During tensile testing, the exterior layers of composites composed of GF may have absorbed the stress and distributed it throughout the composites. Prior to failure, the RS at the core of the composite is subjected to less stress. The tensile properties of a hybrid composite are identified by the amount of fiber present, the arrangement of the individual fibers, and the degree to which the fibers are intertwined, along with the interfacial adhesion that is present between the fiber and the matrix [53,54]. Figure 6c depicts GF fiber fracture (breakage) and matrix cracking. The fiber breaking indicates that the configuration of HRS5 strength increased due to the incorporation of GF with RS in the epoxy composite, which is supported by the results shown in Figure 5. Matrix cracking occurred during the tensile test due to the brittleness of the epoxy resin [23]. Furthermore, based on the fiber-breakage phenomenon, the stress was efficiently transferred from the matrix to the woven fiber. As a result, woven fiber serves as an effective reinforcement in composites. However, in the HRS6 fractured sample with two layers of GF and four layers of RS, an occurrence of whole fiber pull-out was observed. Decreased matrix composition has resulted in weak interfacial adhesion between the fiber and matrix. The woven fiber and matrix, therefore, have poor interfacial adhesion [5]. As a result, our evidence supports the HRS6 sample’s low TS values when compared to those of other samples.

### 3.2. Flexural Properties

**Figure 7:**
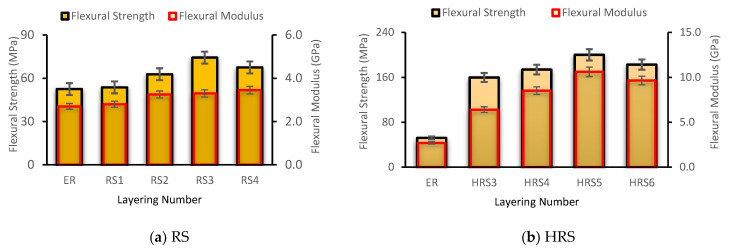
Flexural properties of (**a**) rattan strips (RS) and (**b**) hybrid rattan (HRS) composites.

The flexural characteristics of pure and HRS composites are shown in Figure 7a,b. Similar to TS, the inclusion of a layer of RS significantly boosted the flexural strength of the composites. Flexural modulus and strength rose by up to 22.2% and 41.5%, respectively, as the rattan layer was increased by up to four layers. However, the RS4 result indicates a decrease in flexural strength compared to the RS3 result. Therefore, the results of this study show that compared to virgin ER, RS3 possesses the highest TS and TM (Figure 7a). On top of that, the outcomes of flexural strength and the modulus for the HRS composite sample also present the highest result at HRS5, where the sample consists of two layers of GF and three layers of RS, as shown in Figure 7b. The HRS5 result shows an enhancement of 269% in flexural strength and 321% in flexural modulus, compared with the RS3 samples’ result. This is due to the GF woven located at the bottom and top of the composite’s reinforcement configuration, which is vital in transferring the stress applied to the composite sample. Theoretically, hybrid composite types consist of sandwich, intra-ply, and inter-ply composites. To create lightweight structures, sandwich-type hybrid composites typically consist of two or more distinct layers. This design is commonly employed to support bending loads (the core is constructed of lightweight material, for example, natural fiber, while the skins are composed of high-strength fiber-composite materials) [55,56]. This study shows that the RS composite has a lower strength and stiffness property than the GF composite because it cannot endure the applied load transferred from the epoxy matrix, given the poor interfacial adhesion between the RS and epoxy matrix as well as the RS’s weak nature. Principally, the tensile and compressive conditions of the composite sample are combined to produce flexural behavior [57]. The top surface of the specimen is compressed at the loading point while the bottom surface is under tension. In compression scenarios, the imparted stress is easily transferred from the matrix to the GF across the interface. In the meanwhile, the GF inserted at the bottom of the composite sample successfully strengthened the sample under tension. The flexural modulus and the strength of HRS6 are reduced by 9.64 GPa and 182 MPa, respectively, when the hybrid is constructed with its highest number of layers. This is in line with the findings of most earlier studies [24,58].

### 3.3. Impact Strength

**Figure 8:**
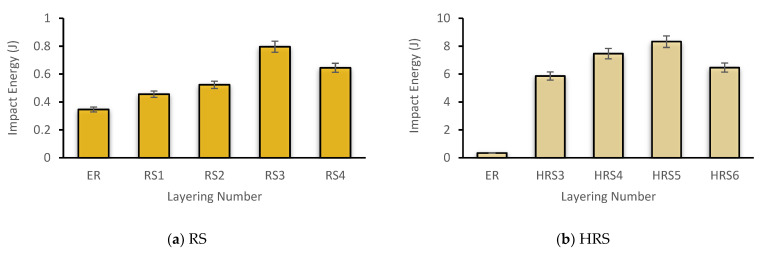
Impact energy of (**a**) pure rattan strips (RS) and (**b**) hybrid rattan (HRS) composites.

Figure 8a,b illustrate the impact strength of pure and hybrid RS composites, respectively. In comparison, the configuration of all pure RS specimens is inferior to that of HRS. RS3 has a maximum impact absorption energy of 0.796 J. For the HRS, HRS5 has the highest impact absorption energy at 8.323 J. Other than that, the impact energy of pure RS had substantially risen when the number of layering configurations was increased from one to three. The maximum stacking concentration of RS4 reduces the impact energy by 0.640 J. A similar pattern was observed for HRS, in which the hybridization of three layers of rattan and two layers of GF at the outer surface significantly improved the maximum impact absorption energy.

**Figure 9:**
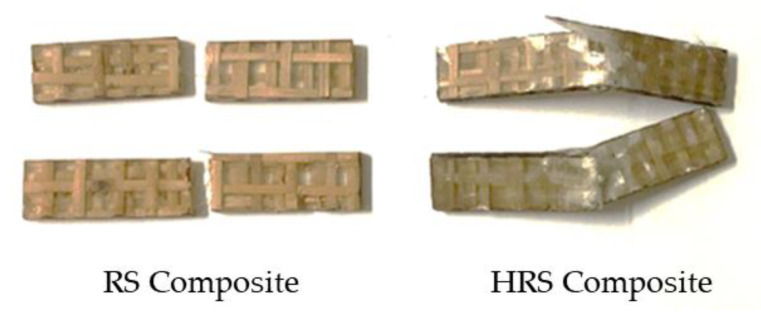
Impact fracture specimens of RS and HRS.

When natural fibers are employed in a hybrid, the polymer composite’s mechanical properties may be strengthened by introducing synthetic fibers, since synthetic fibers help compensate for natural fibers’ limits. In hybrid composites, rattan fiber did not completely fracture because it was bonded between glass fibers (Figure 9). Due to the impact damage, the GF at the top was broken. The fiber cracked at various levels, indicating that a certain amount of energy was absorbed during fiber pull-out. The impact strength findings in this study are consistent with previous studies, which also revealed an increase in impact strength when using a GF hybrid with coir fiber to reinforce polyester composites [59]. Apart from that, the maximum configuration of HRS6’s layering sequence reduced the impact energy to 6.465 J. Similar research by Ramnath et al. (2013) revealed that the impact energies of three-layer hybrids of jute and abaca with two plies of GF are 16 J and 15 J, respectively [24].

### 3.4. Thermogravimetric Analysis

**Figure 10:**
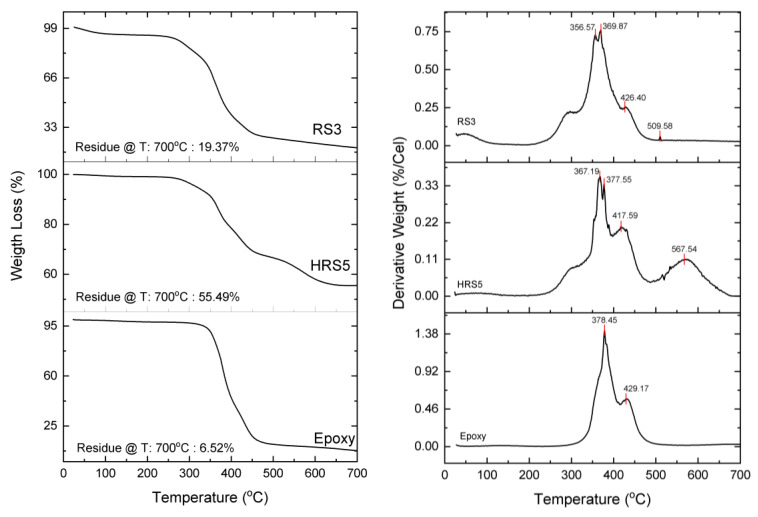
TGA and DTG of virgin epoxy, RS3, and HRS5.

Thermal analysis is a test that can be employed to assess the structural, chemical, and physical changes that occur in a material because of temperature changes. Temperature is an important state variable that influences most structural changes, chemical reactions, and physical qualities [45]. This study observed two different thermal analysis methods: thermogravimetric analysis (TGA) to measure weight loss with temperature change. Figure 10a,b illustrate the graph of thermogravimetric analysis (TGA) and derivative thermogravimetry (DTG) on three samples that were selected based on the mechanical properties of virgin epoxy, RS3, and five layers of HRS with GF composites (HRS5). Thermal decomposition of the composites occurs between 30 and 700 °C. Additionally, Figure 10 portrays the percentage of mass temperature curves, demonstrating that adding pure RS3 and HRS5 in ER decreases the weight loss as a function of temperature. In contrast to other samples, RS3 lost weight at a lower temperature, approximately 4.5% of its initial weight, as shown in the graph, which is comparable to the loss of mass caused by the evaporation of the moisture in the rattan. In the meantime, the virgin epoxy and the HRS5 composite had weight drops of 1.2% and 0.6%, respectively. This is because the hemicellulose in the RS of the composite absorbs more moisture [60]. Figure 10 shows the DTG curves for the epoxy, HRS5, and RS3 samples. It is clear that the main peaks on the DTG curve indicate the maximum decomposition temperature (T_max_), which occurs at temperatures of 378.45, 367.19, and 369.87 °C for the epoxy, HRS5, and RS3 specimens, respectively. T_max_ refers to the degradation temperature showing the maximum weight loss. It also relates to the maximum decomposition temperature. Furthermore, T_max_ denotes an essential indicator showing the material’s thermal stability [31].

The results of this study clearly exhibit the effect of using RS and GF on the stability of epoxy composites, with T_max_ on epoxy being higher than RS3 and HRS5. The DTG curves on HRS5 and RS3 show a small shoulder peak before (200–300 °C) and after (about 500 °C) the main peak or T_max_. These shoulder peaks are usually found in natural fiber composites. Note that the shoulders found between temperatures of 200 °C and 300 °C indicate the presence of hemicellulose, while a temperature of approximately 500 °C can be correlated with lignin decomposition. The decomposition of cellulose from natural fibers occurs at a temperature of about 370 °C, which is associated with the depolymerization and breaking of the epoxy molecular chains (350–400 °C) [61,62]. The findings of this research demonstrate that there is a main peak or T_max_ in the DTG curve, which is almost the same between the fiber and matrix.

Table 3 depicts the weight loss of the epoxy, HRS5, and RS3 by temperature change. Over the temperature range of 200–500 °C, it was possible to notice a mass loss of 73.96% in the RS3 composite. The degradation of the lignocellulosic components of the fiber, for instance, the lignin, hemicellulose, and cellulose constituents, may be responsible for the mass loss that occurs within this temperature range [63,64]. Between temperatures of 200 and 500 °C, virgin epoxy and HRS5 lost approximately 88.56% and 33.56% of their weight, respectively. Based on these observations, it is possible to deduce that adding GF to hybrid composites reduced the decomposition temperature [65]. The addition of GF successfully enhanced the composites’ thermal stability. Identical outcomes were reported for the thermal decomposition of composites made of jute and GF [66]. This result is consistent with previous observations of a similar thermogravimetric trend for composites [67]. At the higher temperature setting of decomposition at 700 °C, it was observed that the char residue of HRS5 composites is the highest compared to those of RS3 and virgin epoxy. According to the findings, while GF hybridization increased both of the hybrid composites’ initial and final decomposition temperatures, their thermal stability improved. Similar conclusions were reported by Ghani et al. [47] in their investigation into the performance of jute/GF with different layering configurations.

### 3.5. Differential Scanning Calorimetry (DSC)

**Figure 11:**
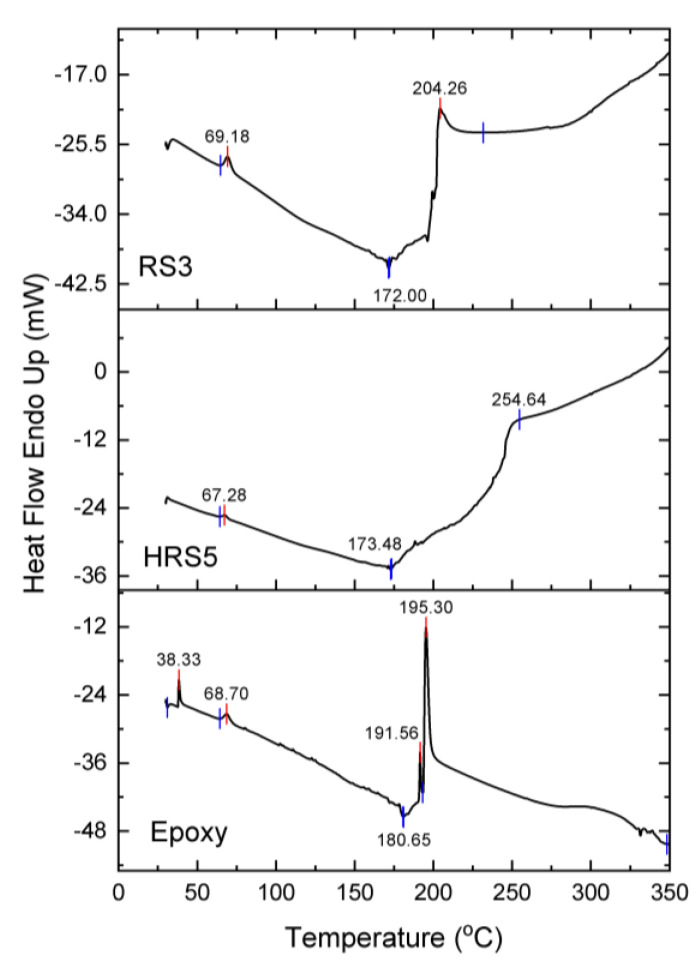
DSC curves of pure epoxy, RS3, and HRS5.

Figure 11 depicts the DSC curve for the epoxy, HRS5, and RS3 specimens. The DSC curve showed the chemical and thermal response of the fibers as the temperature rose. Meanwhile, endothermic peaks in the temperature range of 30 to 175 °C are seen in the HRS5 and RS3 specimens, confirming the existence of water molecules. The occurrence of peaks at 67.28 °C and 173.48 °C in this investigation demonstrated the existence of water molecules in HRS5. The occurrence of peaks at 69.18 °C and 172 °C in RS3 specimens, on the other hand, indicated the existence of water molecules. The epoxy, HRS5, and RS3 specimens were stable between these temperatures, since no endothermic or exothermic reactions were seen in the 80 to 160 °C range [31,68]. Hence, data from the literature indicate that hemicellulose thermal decomposition starts at around 180 °C and concludes at around 350 °C [31,69]. As shown in Figure 11 the decomposition of the hemicellulose on the HRS5 and RS3 occurs at temperatures of 254.46 °C and 204.26 °C, respectively.

## 4. Conclusions

Analysis was done on the thermal and mechanical properties of hybrid and pure epoxy composites reinforced with rattan strips (RS). What happens when glass fiber (GF) is stacked with rattan and hybrid rattan was identified. The tensile, flexural, and impact strengths of a pure rattan-reinforced epoxy composite grew as the stacking number rises, according to the composite’s mechanical properties. In addition, three layers of woven rattan showed the highest mechanical properties when contrasted with the other configurations. The hybrid rattan and GF showed that the combination of three layers of RS and two plies of GF increases the mechanical significantly. The thermal properties of the selected materials, including virgin epoxy, RS3, and HRS5, were assessed by employing thermogravimetric analysis (TGA) and differential scanning calorimetry (DSC). Here, the high decomposition temperature revealed that the RS and GF hybridization is more thermally stable. The hybridization of rattan with GF has effectively enhanced the mechanical properties and thermal properties of the composites compared to pure rattan. Therefore, it is feasible to conclude that hybridization is a method for enhancing the strength, modulus, energy absorption, and thermal stability of polymeric composites. Moreover, the findings of this study indicate that the hybridization of a rattan/GF-reinforced epoxy composite possesses the potential to be utilized in automobile bumper beams.

## Figures and Tables

**Table 1:** Thermal and mechanical properties of epoxy, rattan, and glass fiber [30,31,32,33,34].

Properties	Epikote 828	Rattan	E-Glass Fiber
Density (g/cm^3^)	1.16	0.45	2.55–2.6
Initial viscosity @ 25 °C (Pa s)	<10–12	-	-
Modulus (GPa)	3	4.97	72–85
Tensile strength (MPa)	60	41.97–121.5	1900–2050
Elongation at break (%)	4	-	1.8–4.8
Glass transition (°C)	155	-	-

**Table 2:** Configuration model and label code of specimen test.

Layering Number	Configuration Model and Label Code	
	Rattan Strip	Hybrid Rattan Strip
1	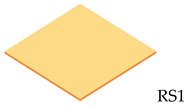	-
2	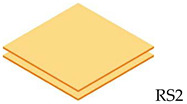	-
3	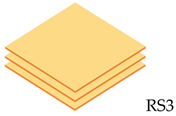	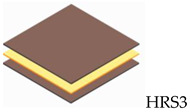
4	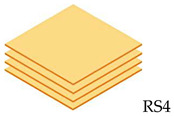	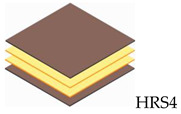
5	-	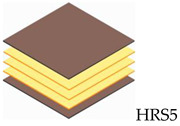
6	-	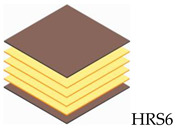

**Table 3:** The weight loss of epoxy, HRS5, and RS3 by temperature change.

Temperature (°C)	Weight Loss (%)		
	Epoxy	HRS5	RS3
25–200	0.95	0.75	5.54
200–500	88.56	33.56	73.96

## Data Availability

Data are contained within the article.
